# Phylogenies and traits provide distinct insights about the historical and contemporary assembly of aquatic insect communities

**DOI:** 10.1002/ece3.2081

**Published:** 2016-03-24

**Authors:** Victor S. Saito, Marcus Vinicius Cianciaruso, Tadeu Siqueira, Alaide A. Fonseca‐Gessner, Sandrine Pavoine

**Affiliations:** ^1^ Programa de Pós‐Graduação em Ecologia e Recursos NaturaisUniversidade Federal de São CarlosSão CarlosSPBrazil; ^2^ Centre d'Ecologie et des Sciences de la Conservation (CESCO UMR7204)Sorbonne Universités, MNHN, CNRS, UPMCCP51, 55‐61 rue Buffon75005ParisFrance; ^3^ Departamento de EcologiaUFG ‐ Universidade Federal de GoiásGoiásBrazil; ^4^ Departamento de EcologiaUNESP – Universidade Estadual PaulistaRio ClaroBrazil; ^5^ Departamento de HidrobiologiaUFSCar ‐ Universidade Federal de São CarlosSão CarlosBrazil; ^6^ Mathematical Ecology Research GroupDepartment of ZoologyUniversity of OxfordOxfordUK

**Keywords:** Assembly rules, community assembly, ecophylogenetics, habitat filtering, niche complementarity, trait structure

## Abstract

The assumption that traits and phylogenies can be used as proxies of species niche has faced criticisms. Evidence suggested that phylogenic relatedness is a weak proxy of trait similarity. Moreover, different processes can select different traits, giving opposing signals in null model analyses. To circumvent these criticisms, we separated traits of stream insects based on the concept of *α* and *β* niches, which should give clues about assembling pressures expected to act independently of each other. We investigated the congruence between the phylogenetic structure and trait structure of communities using all available traits and all possible combinations of traits (4095 combinations). To account for hierarchical assembling processes, we analyzed patterns on two spatial scales with three pools of genera. Beta niche traits selected a priori – i.e., traits related to environmental variation (e.g., respiration type) – were consistently clustered on the smaller scale, suggesting environmental filtering, while *α* niche traits – i.e., traits related to resource use (e.g., trophic position) – did not display the expected overdispersion, suggesting a weak role of competition. Using all traits together provided random patterns and the analysis of all possible combinations of traits provided scenarios ranging from strong clustering to overdispersion. Communities were phylogenetically overdispersed, a pattern previously interpreted as phylogenetic limiting similarity. However, our results likely reflect the co‐occurrence of ancient clades due to the stability of stream habitats along the evolutionary scale. We advise ecologists to avoid using combinations of all available traits but rather carefully traits based on the objective under consideration. Both trait and phylogenetic approaches should be kept in the ecologist toolbox, but phylogenetic distances should not be used as proxies of traits differences. Although the phylogenetic structure revealed processes operating at the evolutionary scale, only specific traits explained local processes operating in our communities.

## Introduction

The understanding of local community assembly advanced substantially when ecologists began to change their focus from pure compositional approaches to those that consider differences among species (Cadotte et al. 2013). Phylogenetic and trait‐based approaches have been proposed as means to provide insights on whether environmental filtering and/or limiting similarity are the main drivers of community assembly (Weiher and Keddy 1995; Webb et al. 2002). The logic of these approaches is to compare observed trait and phylogenetic structures of communities with those expected under null models. When species with similar niches co‐occur more than expected by chance, a trait‐ (or phylogenetic) clustered pattern would indicate the prevalence of environmental filtering (considering niche conservatism in the phylogenetic approach). In the opposite scenario, when species with similar niches co‐occur less than expected by chance, a trait‐ (or phylogenetic) overdispersed pattern would be inferred as limiting similarity excluding similar competing species.

A possible advantage of the phylogenetic approach is that one does not need to select and measure the traits that are important to community assembly (Mason and Pavoine 2013). Instead, it is assumed that these traits are conserved through evolution, and their signal should appear in the phylogenetic structure of local communities (Webb et al. 2002). However, after a plethora of studies, ecologists started debating whether phylogenies are useful to tackle community assembly questions (Mayfield and Levine 2010; Pavoine and Bonsall 2011; Cadotte et al. 2013; Mason and Pavoine 2013; Gerhold et al. 2015). In several scenarios, the phylogenetic structure of a community provides limited power to infer assembly processes even when traits have strong phylogenetic signal (Mason and Pavoine 2013). For example, greater competitive asymmetry among distant relatives (Mayfield and Levine 2010) and facilitation among close relatives (Sargent and Ackerly 2008) can also cause phylogenetic clustering. The phylogenetic structure could thus be better used to tackle other questions rather than used as a proxy of species ecological similarity (Swenson 2013; Gerhold et al. 2015). Phylogenies could give clues about the dispersal limitation of clades (Saito et al. 2015a, 2015b) or reveal the colonization history of habitats (Gerhold et al. 2015; Lososová et al. 2015; Sobral and Cianciaruso 2015). For example, high levels of phylogenetic diversity within communities in comparison to the regional species pool could be interpreted not as a limiting similarity, but as an efficient colonization of distantly related clades from the species pool (Swenson et al. 2012).

Similarly, although the trait‐based approach repeatedly proved its strength to predict local assembly processes (Weiher et al. 2011; Swenson et al. 2012; Kraft et al. 2015), a number of concerns have also been raised. First, some traits are related to competitive interactions while others are more related to habitat filtering providing opposite signals in analyses (Colwell and Winkler 1984). Second, similar to the problem in the phylogenetic approach, competitive exclusion can result in a clustered pattern if assembling traits are related to competitive asymmetry among species (Mayfield and Levine 2010). In this sense, the correct interpretation of clustering or overdispersion is a fundamental part of community assembly studies and requires detailed knowledge of systems and organisms (Mayfield and Levine 2010; Cadotte et al. 2013).

One way to tackle these problems is to search for key traits that are more reasonably linked to specific processes (Ingram and Shurin 2009; Pavoine and Bonsall 2011; Mason and Pavoine 2013; Winemiller et al. 2015). The concepts of the *α* niche and *β* niche (Ackerly and Cornwell 2007) can be used to separate traits that could be expected to respond independently of each other in community assembly. Indeed, *α* niche traits and *β* niche traits should provide opposite signals when tested together against the same null model. Alpha niche traits would be those related to resource use within a community and thus expected to be evenly spaced if competition is a strong driver; and *β* niche traits would be those related to the environment that a species could inhabit and thus expected to be clustered if environmental filters are important.

One way to integrate these approaches is by explicitly recognizing that assembling processes act hierarchically on different spatial scales. During community assembling, environmental filtering is expected to first act and at large spatial levels (Cavender‐Bares et al. 2006; Ackerly and Cornwell 2007) while potential competitors are only those who have already passed this filtering acting thus locally (Götzenberger et al. 2012). The use of different scales and their associated pools of taxa can thus give clues about dispersal limitations that inhibit lineages to co‐occur (Mittelbach and Schemske 2015; Sobral and Cianciaruso 2015). Based on this reasoning, community assembly studies should account for different scales and species pools to properly detect assembling processes.

Inspired by this, we studied stream insect communities because their trait and phylogenetic structure has potential to reveal signals of hierarchical ecological and evolutionary assembling. For example, channel structure and water chemistry are well‐known to be strong forces acting over traits and filtering species in streams (Poff 1997). There is also evidence that competition may be an important driver of insect communities in streams mainly due to exploitative competition for food and space (Miyasaka et al. 2003). Moreover, the idea of hierarchical filters acting subsequently in streams is supported by other studies (Poff 1997). This provides sufficient evidence for us to expect this phenomenon in our system. In addition, the phylogenetic structure of aquatic insects can complement our understanding by shedding light on processes like dispersal limitation of lineages from the species pool (Saito et al. 2015a, 2015b).

Based on this, we tested the following hypotheses related to the community assembly of aquatic insects. (H1) Limiting similarity and environmental filtering are both important drivers of community assembling but are only detectable when analyzing *α* and *β* niche traits, respectively. We predicted that overall trait similarity would show random patterns, while *α* niche traits would be overdispersed within communities, supporting limiting similarity, and *β* niche traits would be clustered, revealing habitat filtering. If limiting similarity and environmental filtering are not strong drivers, then *α* and *β* niche traits should not show patterns different from those expected under the null model. (H2) Assembly processes act hierarchically with environmental filtering acting first and on a larger community scale than competition. We predicted that *α* niche traits would be overdispersed only when communities are considered on the riffle micro‐scale, while *β* niche traits would be clustered only when communities are considered on the stream scale. (H3) Aquatic insects are hypothesized to have colonization limitation with increasing spatial extent. We predicted that communities would have random phylogenetic structures on local community scales, but would show increasing clustering over increasing scales and increasing size of the pool of taxa.

## Material and Methods

### Sampling and study design

The study area is located in the Itanhaém river basin in southeastern Brazil (24°10′58″S, 46°47′20″W). This is a region with subtropical weather with hot summers (28°C average) and mild winters (17°C average). This catchment is located within the littoral of São Paulo State and is characterized by headwaters that range from near pristine to slightly disturbed by banana plantations. The headwaters are slightly acid (pH: 5.1–7.4) with low conductivity (0.023–0.039 *μ*S/cm). The water is formed by many parts of riffles with gravel (65–500 mm) and boulders (>500 mm). There are a few pools with sand and litter. The maximum depth in these headwaters is less than 100 cm and the width ranges from 55 to 363 cm.

**Figure 1:**
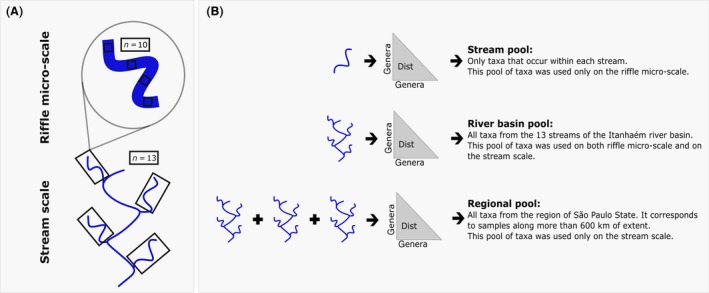
Schematic view of study design and pools of genera used in different null models. (A) On riffle micro‐scale, each sample (30 × 30 cm) was considered an entire community, while, on the stream scale each community was the sum of abundances over the 10 samples collected. (B) We used three different pools of genera in our study. The stream pool is composed of all genera from the same stream as the analyzed sample. It thus considers that interacting genera are only those that inhabit the same stream. The river basin pool considers all genera from the Itanhaém river basin. It does not consider dispersal limitation on the river basin scale. The regional pool considers taxa sampled in the whole São Paulo State region. This last pool is more prone to reveal patterns due to large‐scale processes.

We selected 13 headwater streams and collected 10 samples per stream using a Surber sampler (net mesh size 0.025 mm and 900 cm² area) in riffles for a total of 130 riffles. Our riffle micro‐scale communities were each Surber samples (*n* = 130), and the communities on the stream scale were composed of the sum of 10 samples in the stream (*n* = 13) (see Fig. 1 for a schematic view of sampling design). The insects were screened in vivo using illuminated trays and were preserved in 70% alcohol solution. Most of the insects were identified to the genus level, but some Lepidoptera and Diptera were left at the family level, i.e., Pyralidae, Dyxidae, Chironomidae.

### Aquatic insects supertree

We used the supertree published in Saito et al. (2015a) that contains almost all genera used in our analysis. Genera not contemplated in Saito et al. (2015a) were included as new polytomies because all of their families were present in the former supertree. The original supertree used information on the age of 32 nodes compiled with reference to several recently proposed phylogenies constructed with both morphological and molecular information (see Saito et al. 2015a for details). Branch length were assigned using BLADJ algorithm that spaces undated nodes evenly between dated nodes using and adjuster algorithm (Kembel et al. 2010). To construct a phylogenetic distance matrix, we used the cophenetic distances among genera on the supertree.

### Traits of aquatic insects

To construct trait distance matrices, we used traits available for tropical aquatic insects in the literature (Poff et al. 2006; Tomanova et al. 2006; Colzani et al. 2013). The overall trait distance was calculated with the following traits: voltinism and life span (life cycle traits); exoskeleton, body shape, respiration, BMWP index (Biological Monitoring Working Party), body size, flight capacity and shelter (morphological/physiological traits); and reophily, microhabitat preference and trophic position (behavioral traits) (for a complete description of trait states, see Colzani et al. 2013). Trait values were assigned at the genus level for all traits except for the BMWP index, which is calculated per family as a level of tolerance to pollution. In this latter case, all genera within families were assigned the same BMWP score. To construct trait distances, we used the modified Gower distance because it can handle numerical, categorical and ordinal data (Gower 1971; Pavoine et al. 2009).

### *α* niche traits

The selection a priori of *α* niche traits was based on the four niche axes that determines the strength of species competition (Amarasekare 2003). Species may differ in terms of the resource used (nutrients, food), where they use the resource (space) and when they use this resource (time). A weaker interspecific competition is expected when species differ in the traits related to these niche axes. Thus, if competition is a strong driver in our communities, we expected to find overdispersed patterns in local *α* niche traits when compared to distributions generated at random. For aquatic insects, those selected to compose the *α* niche distance were “reophily”, “microhabitat preference” and “trophic position”. Reophily and microhabitat preferences represent the spatial niche. They indicate in which water velocity and stream substrate the species usually occur. Trophic position is related to the resource use axis because it indicates the foraging strategy of species.

In riverine landscapes, higher environmental heterogeneity is expected within streams (compared to among streams), as indicated by the variation in substrate types, the amount of organic material, depth and width (Heino 2005). This heterogeneity should provide all kinds of microhabitats within each stream (Heino 2005). Each microhabitat can be considered a competing resource, i.e., it can be expected to show overdispersed patterns if competition for microhabitat is intense. For example, hydropsychid congeners can have a segregated distribution at the micro‐scale due to aggressive competition for food and net supplies (Harding 1997).

### *β* niche traits

We selected *β* niche traits a priori as phenotypic traits expected to be linked to physiological limitations in individuals. For example, low levels of dissolved oxygen may limit the establishment of insect larvae in a gradient of river pollution. Following this reasoning, we selected “BMWP index” and “respiration” to compose the *β* niche traits. Thus, if environmental filters were the determinants of community structure, we should find clustered patterns compared to the distribution of traits drawn from a null model.

The BMWP is a biotic index that gives each family a score of tolerance to organic pollution ranging from 1 (very tolerant) to 10 (very sensitive) (Hawkes 1998). The “respiration” trait is composed of three different strategies of oxygen uptake: “tegumental respiration”, “gill respiration”, and “aerial respiration”. A transition from taxa with gill respiration through taxa with cutaneous respiration to taxa with air respiration relying on spiracles, plastrons, or tracheae is expected with increasing environmental harshness (Saito et al. 2015a).

### Phylogenetic signal

We investigated if there was a phylogenetic signal – i.e., the tendency of related genera to resemble one another more than they resemble genera drawn at random from the phylogenetic tree – in different ways. First, we measured phylogenetic signals using Mantel test between the square root of the phylogenetic distances (Hardy and Pavoine 2012) and the trait distances created with each individual trait (12 traits), with combined *α* and *β* traits, and with all traits combined. Phylogenetic signal in nominal traits (shelter, exoskeleton, body shape, reophily, microhabitat preference, trophic position, and respiration) was also tested using Maddison and Slatkin (1999) method, which compares the minimum number of trait changes to a distribution of changes drawn from a null model (Maddison and Slatkin 1991). Ordinal (voltinism, body size and flight capacity) and quantitative (BMWP index) traits were also tested for phylogenetic signal using Blomberg et al. (2003) *K*, K*, and Pavoine and Ricotta (2013) Kw statistics.

Changes in trait states are not necessarily linear over time because they are dependent on different events and pressures along the evolutionary history of a clade (Diniz‐Filho et al. 2010). For example, rapid trait evolution can happen in the beginning of the diversification history of a clade with a posterior period of stasis (Losos 2008), thus we used Mantel correlograms in each individual trait, in combined *α* and *β* niche traits, and in overall trait distance to reveal complex patterns in phylogenetic signal of traits (see Appendix S1).

### Community structure analysis

We calculated metrics of phylogenetic and trait community structure on the two scales (riffle micro‐scale and stream scale) using Mean Nearest Neighbor Distance (MNND), and Mean Pairwise Distance (MPD) (Webb et al. 2002). The MNND metric is calculated as the mean distance to the closest relative individual (or genus when using incidence data) between all individuals (or genus) in a community (Webb et al. 2002). The MPD metric is calculated as the mean phylogenetic or trait distance among all individuals (or genus) in a community. To test whether the phylogenetic and trait structure of the communities were more clustered or dispersed than expected by chance, we used minus the standardized effect size of MNND and MPD, which are called the Nearest Taxon Index (NTI) and Net Relatedness Index (NRI), respectively (Webb et al. 2002). NTI and NRI compare the observed values to null values of MNND and MPD, respectively. Null values were obtained using the null model “taxa shuffle” that randomizes the rows and columns of the matrix of phylogenetic or trait distances among genera 1000 times (Kembel 2009). Following the conceptual approach of null models – fixing all data patterns except the one of interest – we selected the taxa shuffle model because it randomizes only the locations of the taxa in the distance matrix. The model thus constrains the richness and abundance patterns of samples, and allows only the effect of distances to vary (Kembel 2009). This null model can have inflated Type 1 error when abundances of taxa are not randomly distributed across the phylogeny (Hardy 2008) or trait‐based distances. Thus, we used Hardy's (2008) test called ‘Abundance Phylogenetic Deviation’ (APD) to look for ‘abundance phylogenetic clustering or overdispersion’. This test was developed for the phylogenetic approach, but we applied it also with traits by replacing phylogenetic distances in APD with trait distances among species, thus testing for ‘abundance trait clustering or overdispersion’. We did not find evidence for clustering or overdispersion, supporting the use of the taxa shuffle null model in our study (see Appendix S2, Table S3). We decided to use both NTI and NRI indices because NTI is less influenced by higher levels of phylogenetic and trait structure and is expected to have more power to show overdispersion, while NRI captures the whole structure of assemblages and is more robust to detect clustering (Kraft et al. 2007). All analyses were run using incidence data and abundance data because competition is expected to be density‐dependent, while some physiological constraints could act at the species, here genus, level (Swenson et al. 2012). All analyses were run with phylogeny, all combined traits, *α* niche traits and *β* niche traits. To test for clustering versus overdispersion of communities, we applied the two‐tailed Wilcoxon tests (assuming significance for *P *<* *0.01) in NTI and NRI results. If NTI or NRI were lower than zero, we inferred an overall tendency to overdispersion. The opposite would mean an overall tendency to clustering (Webb et al. 2002).

We used three different pools of aquatic insect genera in null model randomizations that represent distinct hypothetical scenarios. (1) The river basin pool was composed of taxa found in the 13 streams of the Itanhaém river basin. The null model using the river basin pool of taxa assumes that the trait and phylogenetic structure of the 13 communities are not influenced and cannot be colonized by genera from outside the Itanhaém river basin. It also assumes that there is no dispersal limitation among streams of the Itanhaém basin. (2) The regional pool encompasses additional taxa (*n* = 160) found in the region of São Paulo State (samples from the whole State, Suriano et al. 2011) and considers that genera in the Itanhaém river basin could already be a subsample of the regional pool due to large‐scale processes (Mittelbach and Schemske 2015 and see “Discussion”). (3) The stream pool considers only genera from the same stream. This pool assumes short dispersal limitations and assumes that possible interacting genera are only those occupying the same stream (Fig. 1). The river basin pool (1) was used for analyses on both the riffle micro‐scale and the stream scale. The regional pool (2) was used only on the stream scale, and the stream pool (3) was used only on the riffle micro‐scale. Results of the regional and the stream pools can be found in Appendix S2.

To investigate the influence of trait selection in the output of NTI and NRI, we ran analyses using all possible combinations of the 12 selected traits (4095 combinations). For brevity, in these investigations we ran 200 randomizations for NTI and NRI. The influence of trait selection was investigated using the river basin pool of taxa for both the riffle micro‐scale and stream scale, with both incidence and abundance data. To summarize the results using all combinations of traits, we used a redundancy analysis (RDA) with the results of all indices as response matrix and the composition of each of the 4095 combinations of traits as explanatory matrix. The RDA shows which traits are positively or negatively associated with the results of NTI and NRI using all possible combinations of traits. Analysis of NRI and NTI using individual traits were also ran, but it provided similar patterns as those explored through the RDA, thus for brevity these results are presented in Appendix S3.

All analyses were run in R using packages *ade4* (Chessel et al. 2004) and *picante* (Kembel et al. 2010).

## Results

*α* and *β* niche traits, and all traits together showed significant phylogenetic signal according to the Mantel test (Table 1). However, besides the phylogenetic signal in all traits together (Mantel correlation: *r* = 0.63, *P *=* *0.001), the phylogenetic signal in *α*,* β* and individual traits were low (between *r* = 0.38, *P *=* *0.001 and *r* = 0.17, *P *=* *0.005). Among the individual traits, most of them presented phylogenetic signal according to Mantel test, unless respiration (*β* niche trait), shelter and reophily (*α* niche traits) (Table 1). Blomberg's *K*, K* and Kw presented similar results; all resulted in significant phylogenetic signal in ordinal and quantitative traits. For brevity, we present here only the results of K* (Table 1), but results of *K* and Kw can be found in Appendix S1 (Table S1). Phylogenetic signals in nominal traits using Maddison and Slatkin (1999) method were also significant for most of traits (Table 1), except reophily. An explanation for distinct results using Mantel tests and Maddison and Slatkin (1991) approach can be found in Appendix S1.

**Table 1:** Testing the phylogenetic signal of aquatic insect traits using three tests: Mantel test between the square root of the phylogenetic distance and the trait‐based distance, Blomberg et al. (2003) K* for ordinal (rank‐transformed) and quantitative traits, and Maddison and Slatkin (1991) method for nominal traits. Alpha niche traits are reophily, micro habitat preference and trophic position; *β* niche traits are respiration and the BMWP index

	Mantel *r*	Mantel *P*	K* *P*	Maddison and Slatkin *P*	Data type	Trait group
All traits	0.63	**0.001**	**–**	**–**	Multiple traits	Group of traits
*α* niche traits	0.38	**0.001**	**–**	**–**	Multiple traits	Group of traits
*β* niche traits	0.17	**0.005**	**–**	**–**	Multiple traits	Group of traits
Voltinism	0.25	**0.001**	**0.001**	**–**	Ordinal	Life cycle
Adult life span	0.40	**0.001**	**–**	**0.001**	Nominal	Life cycle
Exoskeleton	0.27	**0.001**	**–**	**0.001**	Nominal	Morphology/physiology
Body shape	0.26	**0.001**	**–**	**0.001**	Nominal	Morphology/physiology
Respiration	0.09	0.060	**–**	**0.001**	Nominal	Morphology/physiology
BMWP	0.14	**0.001**	**0.001**	**–**	Quantitative	Morphology/physiology
Body size	0.27	**0.001**	**0.001**	**–**	Ordinal	Morphology/physiology
Flight capacity	0.21	**0.001**	**0.001**	**–**	Ordinal	Morphology/physiology
Shelter	0.05	0.110	**–**	**0.001**	Nominal	Behavioral
Reophily	0.09	0.034	**–**	0.199	Nominal	Behavioral
Micro habitat	0.27	**0.001**	**–**	**0.001**	Nominal	Behavioral
Trophic position	0.20	**0.001**	**–**	**0.001**	Nominal	Behavioral

Significant values are in bold.

Most Mantel correlograms did not show regularly decreasing curves indicating that the evolution rate in traits was not constant throughout the phylogenetic tree. The distances calculated on all traits together and *α* niche traits showed a more linear decrease than *β* niche traits (see Figure S9, Appendix S1). However, absolute correlations were low (<0.32) indicating trait lability.

**Figure 2:**
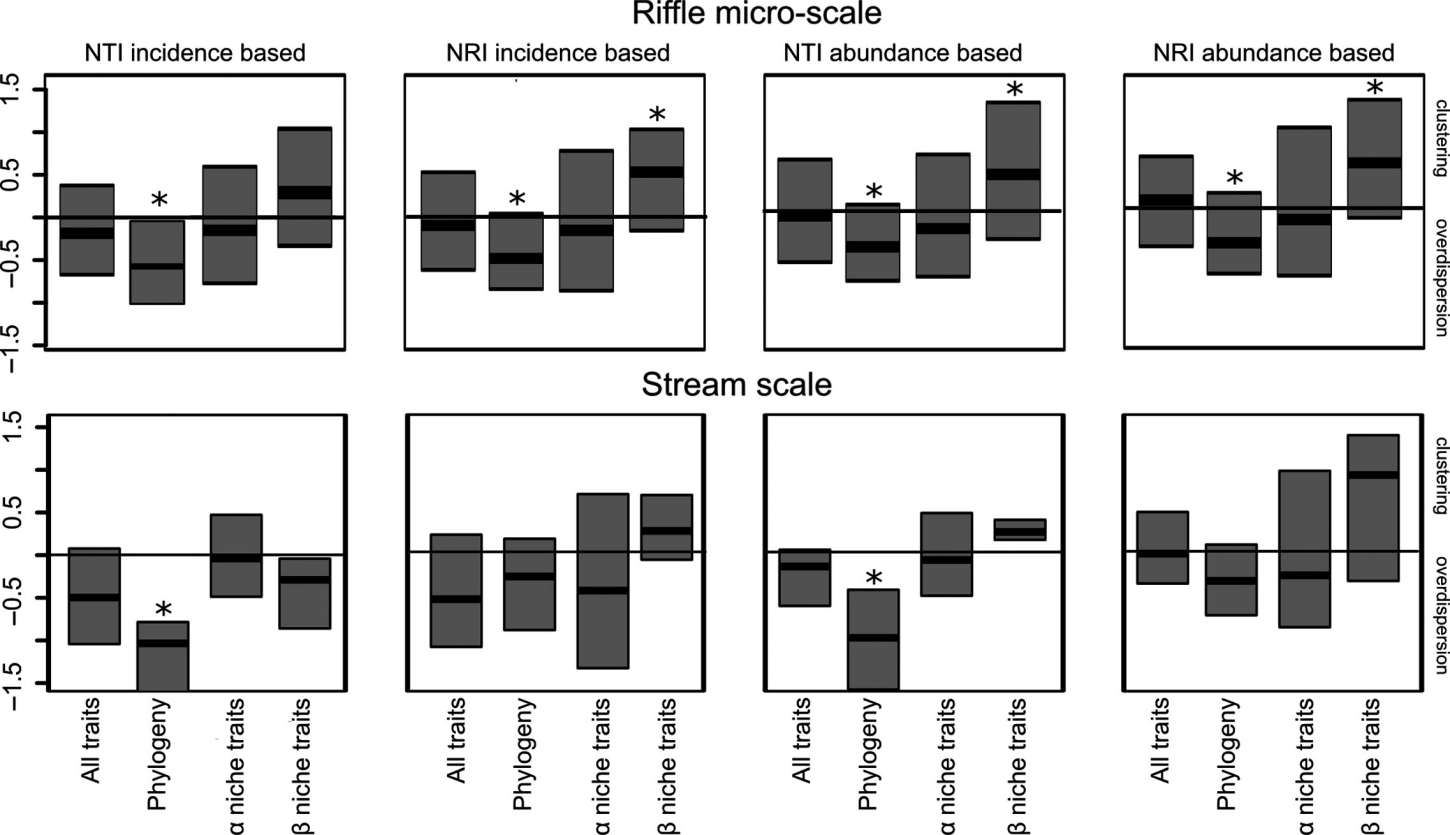
Box plots of values of Nearest taxon index (NTI) and Net relatedness index (NRI) on riffle micro‐scale and on stream scale calculated with trait and phylogenetic distances. The pool of genera used in the null model was composed of taxa found in the 13 streams of the Itanhaém river basin. Trait distances were calculated in three different ways: using all traits, using *α* niche traits and using *β* niche traits. Median values significantly different from zero according to two‐tailed Wilcoxon test have “*” for *P* < 0.01.

For the riffle micro‐scale, using the river basin pool, NTI and NRI computed with all traits and *α* niche traits indicated random patterns – i.e., the results were not different from those expected under random assembly. The *β* niche traits showed clustering in NTI and NRI indicating that local communities are composed of a subset of *β* niche trait states different from one drawn at random (Wilcoxon test, *P *<* *0.01, except for NTI with incidence data where Wilcoxon test was marginally significant, *P *<* *0.02) (Fig. 2). So, our hypothesis H1 was only partially supported since we found evidences for environmental filtering (clustering in *β* niche traits) but not of competition (random *α* niche traits) structuring our communities. In contrast, the phylogenetic structure was significantly overdispersed in both NTI and NRI analyses indicating that co‐occurring genera have distinct evolutionary history (Wilcoxon test, *P *<* *0.01). These results were consistent for both incidence and abundance data (Fig. 2). The results using the stream pool also showed qualitatively similar patterns (Figure S10, Appendix S2). The exception was for phylogenetic NRI (incidence data) that showed clustering; but NTI instead confirmed the overdispersion trend (Figure S10, Appendix S2).

For the stream scale, using the river basin pool of genera, NTI and NRI indicated random patterns for overall trait distance and *α* and *β* niche traits (Fig. 2). Thus, our results do not support our hypothesis H2 that predicted a hierarchical assembly with clustering in *β* niche traits on the stream scale. The phylogenetic structure was consistent with the results found on the riffle micro‐scale, showing overdispersion in NTI and NRI with both incidence and abundance data (Fig. 2). We found similar trends with the regional pool (Figure S11, Appendix S2). The absence of increasing clustering in the phylogenetic structure of communities suggests that phylogenetic dispersal limitation is not acting (refuting our hypothesis H3). One distinct result was a significant clustering in *α* niche traits in NRI with incidence data (Wilcoxon test, *P *=* *0.01). This reinforces the lack of limiting similarity for competitive traits. However, *β* niche traits did not show consistent clustering even when considering all taxa in the region.

**Figure 3:**
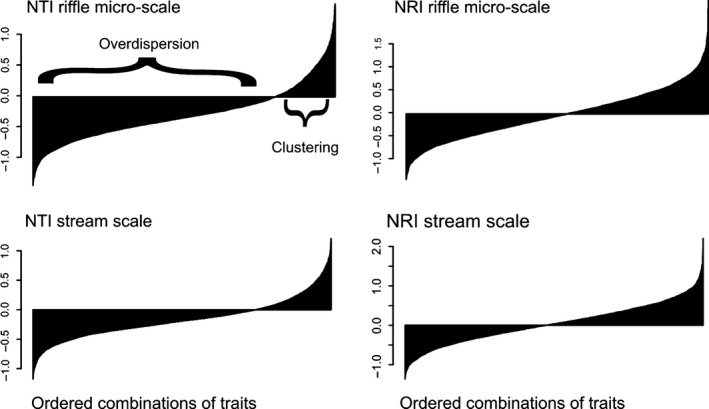
Barplot showing the ordered median value of Nearest taxon index (NTI) and Net relatedness index (NRI) on the riffle micro‐scale and on stream scale using all combinations of traits (*n* = 4095 combinations) and abundance data and considering all genera from the data set as the pool of taxa for the null model. NTI and NRI results are ordered from highest overdispersion to highest clustering.

**Figure 4:**
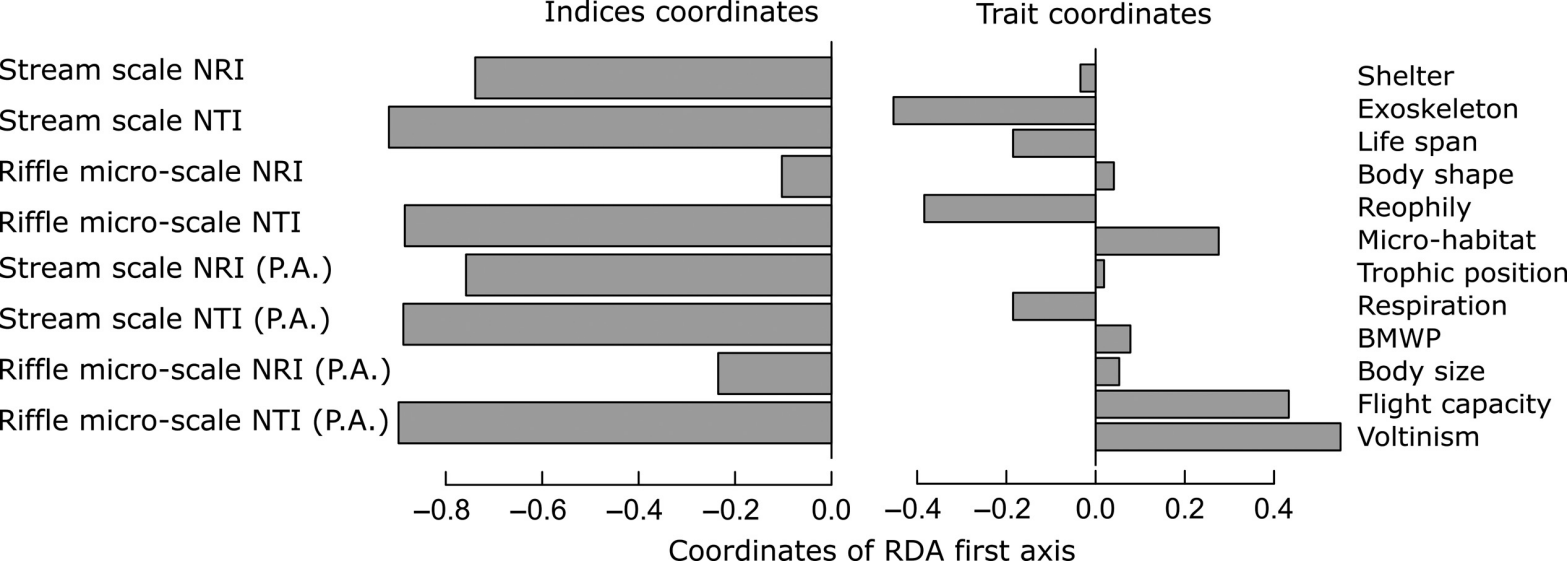
Coordinates in the first axis of redundancy analysis. The response matrix is the result of all indices using all combinations of traits (P.A. means presence–absence data). The explanatory variables were the identities of the traits in the 4095 combinations. The first axis summarizes 87% of variation in the data. Negative coordinates of NRI and NTI on the first axis correspond to high NRI and NTI values and were thus interpreted as trait clustering. NRI, Net relatedness index; NTI, Nearest taxon index.

Against our predictions, calculations of NTI and NRI using all possible combinations of traits did not result in random patterns. Rather, it resulted in values ranging from strong overdispersion to strong clustering depending on the trait combination (Fig. 3). We found that the first axis of RDA summarized a large proportion of variation in the response matrix (87%). This axis showed that exoskeleton, life span, reophily (*α* niche trait) and respiration (*β* niche trait) were associated to clustering results in most of indices (NRI and NTI on the two scales), while microhabitat preference (*α* niche trait), flight capacity and voltinism were associated to overdispersion results (Fig. 4). We did not find consistent evidence for the idea that traits associated to clustering or overdispersion were those related to the *α* and *β* niche of aquatic insects. We found that traits not expected a priori to be structured by competition or environmental filtering were the most related to clustering (exoskeleton) or overdispersion (voltinism) patterns.

## Discussion

Species in a given habitat must share similar traits that enable them to support the same abiotic and biotic pressures including environmental gradients and predators, but they should also be dissimilar in other traits to lower interspecific interference and avoid competitive exclusion (Chesson 2000). However, identifying which trait has strong net effects in which assembly process is not a simple task. The concepts of the *α* and *β* niches (Ackerly and Cornwell 2007) were useful here because they provided a guideline to select traits that are reasonably related to different assembly processes. Using this approach, we found signals that only environmental filtering (clustering in *β* niche traits), but not limiting similarity (consistent random patterns in *α* niche traits), is an important process acting on studied communities. Previous studies that did not separate *α* from *β* niche traits could not disentangle interacting processes. For example, trait clustering using all traits could be due to environmental filtering or asymmetric competition. Moreover, analyses using all possible combinations of traits demonstrated how several, distinct combinations could provide strong clustering or overdispersion, even if they were composed by traits not expected a priori to be related to any assembly process. Our results suggest that using traits without prior expectation can give uninformative results and lead researchers to erroneous inferences.

We expected that *β* niche traits would be clustered within the streams if environmental filtering was strong enough to exclude taxa with inappropriate trait state to occur in a given community. We found random and opposed patterns on the stream scale using the two pools of genera, but consistent clustering on the riffle micro‐scale. This indicates that environmental filtering is a strong driver on the riffle micro‐scale, but not on the (a priori expected) stream scale in the studied communities. The environmental pressures over *β* niche traits were probably not strong enough on the stream scale because we did not design our study along an environmental gradient. Rather, each stream represented a replicate of each other. For the riffle micro‐scale, however, we found that genera with the same respiration strategy and with the same BMWP score tended to co‐occur in small patches of habitat. The BMWP index was created to represent the degree of resistance of aquatic insect families to organic pollution based on the occurrence of taxa along a gradient of impact (Hawkes 1998). This means that families that co‐occur in environments with similar levels of pollution would share the same BMWP score. A gradient of pollution generally follows environmental changes along the entire stream. In the Atlantic Forest biome, a common environmental gradient starts with forested shaded streams with fast waters and rocky streambed. It then passes to streams with lower velocity and with sandier substrate, finally ending in muddy streams with open forest canopy. This suggests that genera with similar BMWP scores should also share strategies to inhabit similar riffle environments. For example, *Gripopteryx* (Plecoptera) and *Farrodes* (Ephemeroptera) have the same score and are dorsoventrally flattened with a similar body size and take oxygen by gills (Pastuchová et al. 2008). In this sense, clustering in the *β* niche traits could be found when species co‐occur in microsites with high oxygenation and without large accumulation of organic matters, as in the small cascades formed by boulders. The results of *β* niche traits support the body of evidence that suggests environmental variation within streams as more important than the variation among streams for structuring aquatic insect communities in nonimpacted streams (Heino et al. 2004; Costa and Melo 2007). Together with results of *α* niche traits, we have indications that aquatic insects with similar micro‐scale preferences usually co‐occur without competitive exclusions. A simple explanation for a relaxed or even clustered co‐occurrence of competition traits (using the regional pool) is that high levels of productivity in a given habitat can maintain high levels of niche overlap without strong competition (Safi et al. 2011). Probably, the high amount of detritus continually entering tropical stream systems over the year enables high feeding overlap among aquatic insects without strong competition among them (Tomanova et al. 2006).

Our findings using all possible combinations of traits suggest that statistically significant clustering or overdispersion can be found using different combinations of traits, with different numbers of traits (see Appendix S3). In many cases, traits strongly associated with clustering or overdispersion were not those related to what we defined a priori as related to the *α* niche and *β* niche of aquatic insects. Some nonexclusive explanations for these results are: (1) we know little about the ecology of aquatic insects in such a way that we did not select all traits that are indeed related to *α* and *β* niche. In this case, the association of some traits, as exoskeleton and voltinism, with clustering and overdispersion is due to ecological processes poorly understood. Although this view looks appealing, it relies on the strong assumption that environmental filtering and competition are always acting over communities; hence one just need to find which traits are responding to these processes. (2) The traits related to clustering or overdispersion actually reflect assembly processes that act in other traits which they are correlated to (e.g., due to phylogenetic or physiological constraints). However, we did not find strong associations between pairs of traits we considered (Mantel *r* < 0.52, Figure S14, Appendix S3), and thus we do not have evidence to support this reasoning. (3) Due to the large number of traits which resulted in an even larger number of trait combinations, there is an increased probability to find patterns of clustering or overdispersion in traits by chance but with few or no possible biological interpretations. Due to the unexpected patterns in several traits (e.g., clustering in exoskeleton and life span and overdispersion in flight capacity and voltinism), we point out this explanation as the most likely in our case study. Nevertheless, these explanations are nonexclusive and we would greatly benefit from further studies using the *α* and *β* niche approach in other ecosystems.

Regarding the phylogenetic structure of aquatic insects, we found consistently overdispersed patterns within communities; a pattern that was commonly used to infer phylogenetic limiting similarity when species present conserved niches (Webb et al. 2002; Cavender‐Bares et al. 2004; Violle et al. 2011). However, the phylogenetic limiting similarity logic can be biased because niche conservatism at the species pool level cannot predict the phylogenetic signal at the community and metacommunity levels (Mason and Pavoine 2013). Simulations showed that local communities composed of close‐related species are not necessarily composed of ecologically similar species, even when evolution of traits is highly conserved at the species pool level (Mason and Pavoine 2013). Although these authors did not make a definitive conclusion, no other study has yet shown opposite results. Consequently, local phylogenetic overdispersion coupled with phylogenetic signal at the pool of taxa level is likely weak evidence of limiting similarity structuring communities.

Following this, alternative explanations for nonrandom phylogenetic structures have recently been developed without relying on the phylogenetic‐patterns‐as‐proxy‐of‐traits approach (Gerhold et al. 2015; Lososová et al. 2015). The phylogenetic structure of local communities is likely influenced by the diversification and dispersal history of lineages as well as by the stability and geological age of the habitat under study (Lessard et al. 2012). A long period of diversification in a given habitat can make contemporary communities share species from very distantly related lineages. Short‐term adaptation and diversification would make co‐occurring species only share a small amount of history (Lososová et al. 2015). In this sense, although local contemporary communities are ephemeral, habitat types, such as streams, are available for colonization and evolution for a long time (Pauls et al. 2006). This makes local communities a result of lineage‐diversification over millions of years (Gerhold et al. 2015). Due to physical and chemical characteristics of water, fast flowing streams were more stable environments for diversification and less prone to entire clade extinctions than terrestrial habitats (Ross 1967). For example, stream‐dwelling insects used fast flowing mountainous streams (that were not frozen) as refuge during glaciations, avoiding regional extinctions (Pauls et al. 2006). Also, an initial colonization and diversification of several aquatic insect orders in oxygen‐rich, cool‐water streams was hypothesized (Ross 1967), which would explain the presence of ancient families in these habitats. Moreover, tropical lineages of aquatic insects probably suffered less extinction events due to less severe effects of glaciation in the Pleistocene. This enables survivorship of relictual taxa of some orders (De Moor and Ivanov 2008). Some of these relictual lineages have widespread and common co‐occurring genera in our study region such as *Chimarra* (Philopotamidae, Trichoptera) and *Beatis* (Baetidae, Ephemeroptera) (De Moor and Ivanov 2008). In this way, the co‐occurrence of these genera would generate high values of phylogenetic diversity within a community because they diverged close to the root of our supertree. This can explain the phylogenetic overdispersion in local communities and corroborate findings of high phylogenetic diversity within (Figure S12, Appendix S2) and among streams for several groups of aquatic insects (Saito et al. 2015b). Thus, phylogenetic overdispersion and high local phylogenetic diversity—combined with low phylogenetic signal in most traits—are likely the result of the widespread distribution and co‐occurrence of species from groups with long divergence times.

In conclusion, we found signals of assembly processes using an a priori selection of *α* niche and *β* niche traits that were not found using all traits together or using phylogenetic information. We suggest ecologists to avoid using combinations of traits without careful selection based on *α* and *β* niche concepts or any other grouping that make sense for the objective under consideration (e.g., Winemiller et al. 2015). Although assembly processes are difficult to predict they are more likely to be revealed if they are important drivers and if the selection of traits for an analysis relies on robust theory. Previous studies suggested that adding more traits likely increase the way in which a species could be ecologically different from another one, strengthening the power of null model analysis to detect assembly processes when community membership is determined by multiple traits (Kraft et al. 2007). However, our analysis of all combinations of traits shows that the conclusions depend on the selected traits. Adding a trait may change conclusions from overdispersion to randomness or clustering. Thus, ecologists cannot avoid the challenge of trait selection to properly identify assembly mechanisms. In addition, the trait and phylogenetic approaches should be kept together in the toolbox of ecologists because they offer complementary information about community assembling. While trait approaches provide insights about local processes such as habitat filtering, the phylogenetic structure of communities can reveal the signature of processes that work on an evolutionary scale including diversification in ancient habitats.

## Data Accessibility

We agree to archive the data associated with this article.

## Conflict of Interest

None declared.

## Supporting information


**Appendix S1**. Supplementary analyses of phylogenetic signal.
**Table S1.** Result of tests of phylogenetic signal using Blomberg et al. *K* and Kw statistic for ordinal and quantitative traits.
**Table S2.** Results from analysis of phylogenetic signal in individual traits.
**Figure S1.** Figures showing the result of Maddison and Slatkin (1999) method for calculating phylogenetic signal in the shelter trait.
**Figure S2.** Figures showing the result of Maddison and Slatkin (1999) method for calculating phylogenetic signal in the exoskeleton trait.
**Figure S3.** Figures showing the result of Maddison and Slatkin (1999) method for calculating phylogenetic signal in the life span trait.
**Figure S4.** Figures showing the result of Maddison and Slatkin (1999) method for calculating phylogenetic signal in the body shape trait.
**Figure S5.** Figures showing the result of Maddison and Slatkin (1999) method for calculating phylogenetic signal in the reophily trait.
**Figure S6.** Figures showing the result of Maddison and Slatkin (1999) method for calculating phylogenetic signal in the micro‐habitat preference trait.
**Figure S7.** Figures showing the result of Maddison and Slatkin (1999) method for calculating phylogenetic signal in the trophic position trait.
**Figure S8.** Figures showing the result of Maddison and Slatkin (1999) method for calculating phylogenetic signal in the respiration trait.
**Figure S9.** Mantel correlograms showing the correlation among trait distances and phylogenetic distances.
**Appendix S2.** Supporting results from community structure analyses.
**Table S3.** Results of APD test for species abundance phylogenetic and trait clustering or overdispersion.
**Figure S10.** NTI and NRI results on rifle micro‐scale using the two distinct null models.
**Figure S11**. NTI and NRI results on stream scale using the two distinct null models.
**Figure S12.** Relationship between phylogenetic diversity and phylogenetic structure metrics.
**Appendix S3.** Additional analyses exploring the combination of traits.
**Figure S13.** Results of NRI and NTI for each individual trait.
**Figure S14.** The correlations among traits were tested using Mantel correlations.
**Figure S15.** Results of NRI and NTI with a varying number of traits used in distance calculations.
