# Supplementary material for: Phylogenies and traits provide distinct insights about the historical and contemporary assembly of aquatic insect communities
Source: Ecol Evol. 2016 Mar 24;6(9):2925–37. doi: 10.1002/ece3.2081 (PMC4863016; doi:10.1002/ece3.2081)
Supplement: Supplementary file 1 — Appendix S1. Supplementary analyses of phylogenetic signal. Table S1. Result of tests of phylogenetic signal using Blomberg et al. K and Kw statistic for ordinal and quantitative traits. Table S2. Results from analysis of phylogenetic signal in individual traits. Figure S1. Figures showing the result of Maddison and Slatkin (1999) method for calculating phylogenetic signal in the shelter trait. Figure S2. Figures showing the result of Maddison and Slatkin (1999) method for calculating phylogenetic signal in the exoskeleton trait. Figure S3. Figures showing the result of Maddison and Slatkin (1999) method for calculating phylogenetic signal in the life span trait. Figure S4. Figures showing the result of Maddison and Slatkin (1999) method for calculating phylogenetic signal in the body shape trait. Figure S5. Figures showing the result of Maddison and Slatkin (1999) method for calculating phylogenetic signal in the reophily trait. Figure S6. Figures showing the result of Maddison and Slatkin (1999) method for calculating phylogenetic signal in the micro‐habitat preference trait. Figure S7. Figures showing the result of Maddison and Slatkin (1999) method for calculating phylogenetic signal in the trophic position trait. Figure S8. Figures showing the result of Maddison and Slatkin (1999) method for calculating phylogenetic signal in the respiration trait. Figure S9. Mantel correlograms showing the correlation among trait distances and phylogenetic distances. Appendix S2. Supporting results from community structure analyses. Table S3. Results of APD test for species abundance phylogenetic and trait clustering or overdispersion. Figure S10. NTI and NRI results on rifle micro‐scale using the two distinct null models. Figure S11. NTI and NRI results on stream scale using the two distinct null models. Figure S12. Relationship between phylogenetic diversity and phylogenetic structure metrics. Appendix S3. Additional analyses exploring the combination of traits. Figure S13. [file ECE3-6-2925-s001.docx]

**Phylogenies and traits provide distinct insights about the historical and contemporary assembly of aquatic insect communities**

Victor S. Saito, Marcus Vinicius Cianciaruso, Tadeu Siqueira, Alaide A. Fonseca-Gessner, Sandrine Pavoine

**Supporting Information**

**Appendix S1: Supplementary analyses of phylogenetic signal**

We present here the results of analysis of phylogenetic signal described in the main text, but included in Supporting Information for brevity. For ordinal and quantitative traits we run Blomberg’s K (Blomberg, Garland & Ives 2003) and a recent variation Kw (Pavoine & Ricotta 2012). For nominal traits we measured phylogenetic signal using Maddison & Slatkin (1991) method of ‘fixing tree and reshuffling trait states randomly’. We used function ‘phylo.signal.disc’ in R environment, developed by Enrico Rezende (Universidad Autònoma de Barcelona). This function was used by several papers measuring phylogenetic signal in discrete traits (Valiente-Banuet & Verdú 2007; Verdú & Pausas 2007; Bauer *et al.* 2012; Montesinos-Navarro *et al.* 2012; Nichols *et al.* 2013; Moro *et al.* 2015). This test is based on the minimum number of character state changes across the tree based on parsimony reconstructions (Maddison & Slatkin 1991). If related species have similar trait states, the number of changes will be lower than expected based on the null model (Maddison & Slatkin 1991). The minimum number of changes was compared to a distribution of 999 random numbers of changes, which were obtained by swapping the trait states across the tips of the tree.

To observe non-linear patterns in trait evolution we used Mantel correlograms in each individual trait, in combined α and β niche traits, and in overall trait distance. This test would reveal complex patterns in phylogenetic signal of traits.

**Table S1.** Result of tests of phylogenetic signal using Blomberg et al. K and Kw statistic for ordinal and quantitative traits.

|  | K statistic | Kw statistic |
| --- | --- | --- |
|  | *P* value | *P* value |
| Voltinism | 0.001 | 0.001 |
| BMWP | 0.001 | 0.001 |
| Body size | 0.001 | 0.001 |
| Flight capacity | 0.001 | 0.001 |

Most of traits presented phylogenetic signal according to Maddison and Slatkin method. The only exception was reophily.

**Table S2.** Results from analysis of phylogenetic signal in individual traits. The P value is related from comparison to a distribution of number of changes generated by swapping the trait states across the tips of the tree 999 times (Maddison & Slatkin 1991).

| Trait | Nº of trait states | Observed nº of changes | Mean null nº of changes | *P* |
| --- | --- | --- | --- | --- |
| Shelter | 3 | 2 | 11 | 0.001 |
| Exoskeleton | 3 | 4 | 21 | 0.001 |
| Life span | 3 | 6 | 21 | 0.001 |
| Shape | 2 | 3 | 17 | 0.001 |
| Reophily | 3 | 15 | 17 | 0.199 |
| Micro-habitat | 5 | 13 | 18 | 0.001 |
| Trophic position | 5 | 12 | 25 | 0.001 |
| Respiration | 3 | 6 | 13 | 0.001 |


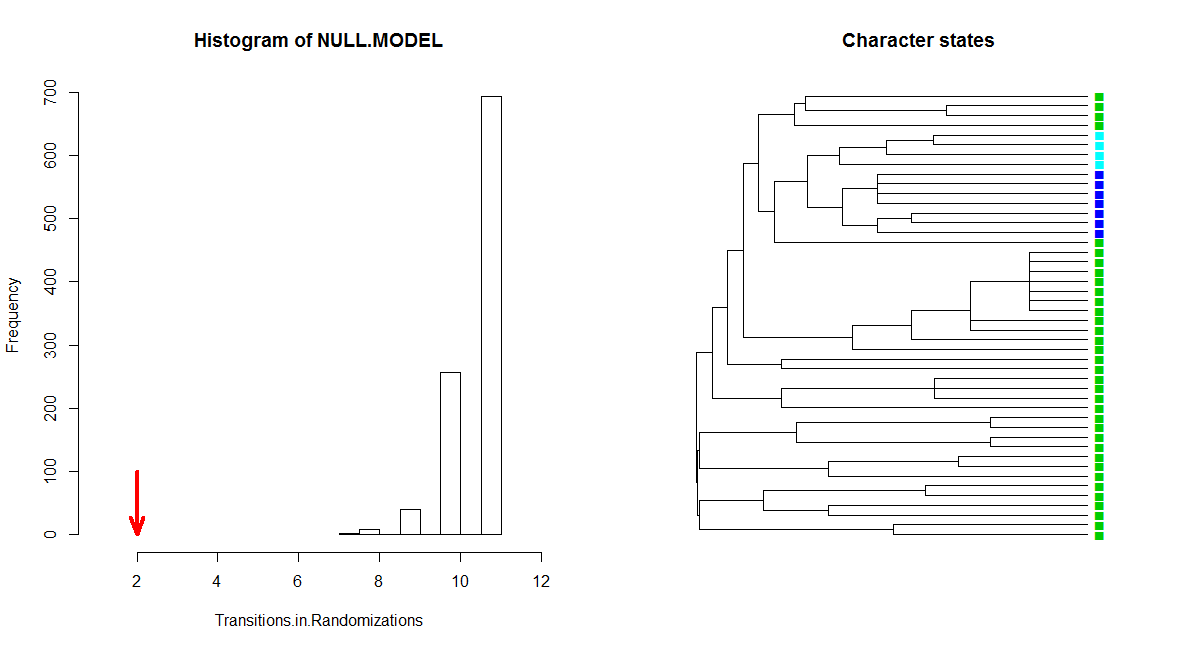


**Figure S1.** Figures showing the result of Maddison and Slatkin (1999) method for calculating phylogenetic signal in the shelter trait. On the left the histogram of null number of changes and the observed number of changes (red arrow); and on the right the phylogenetic tree with each color on the tip showing different trait states.


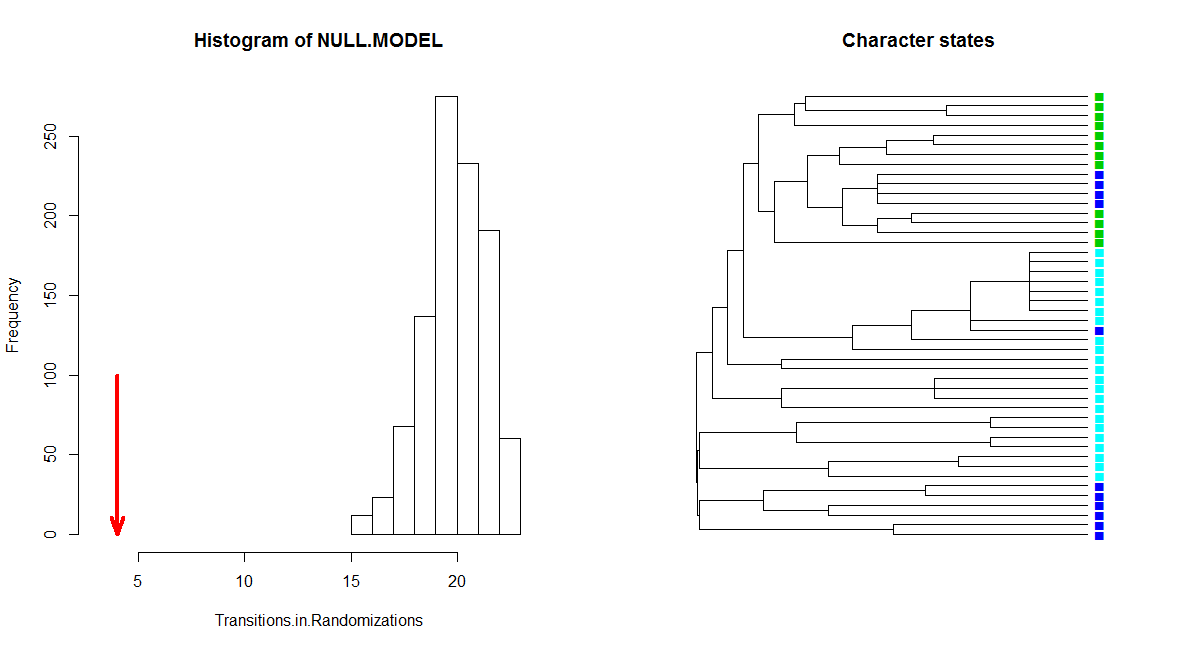


**Figure S2.** Figures showing the result of Maddison and Slatkin (1999) method for calculating phylogenetic signal in the exoskeleton trait. On the left the histogram of null number of changes and the observed number of changes (red arrow); and on the right the phylogenetic tree with each color on the tip showing different trait states.


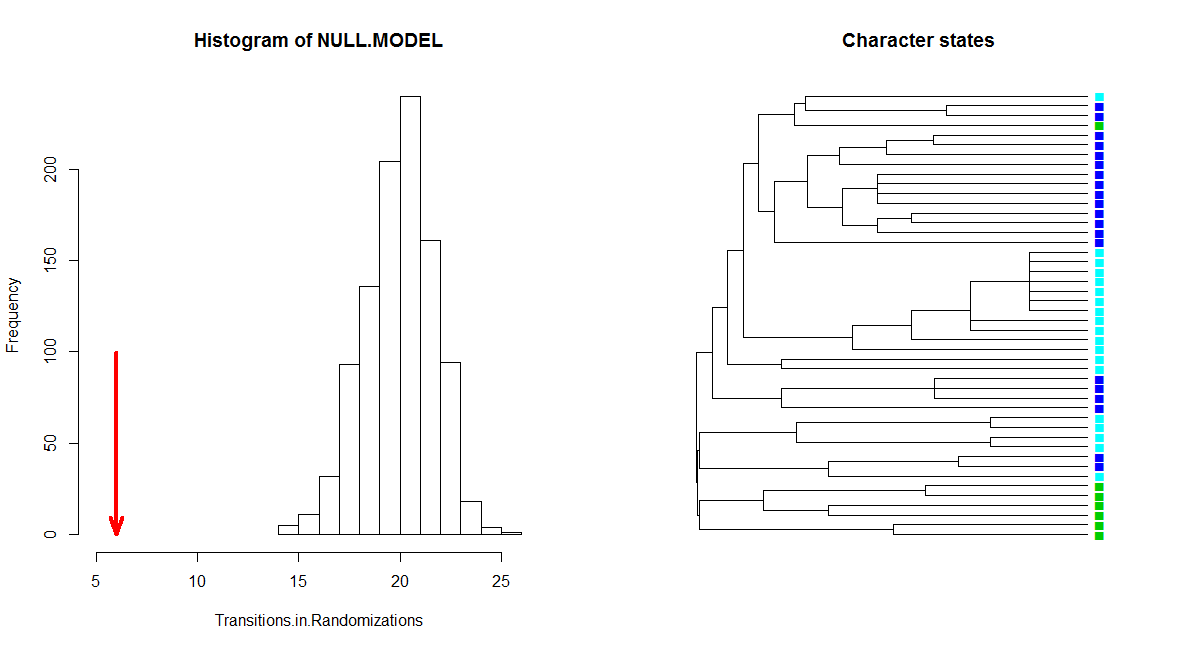


**Figure S3.** Figures showing the result of Maddison and Slatkin (1999) method for calculating phylogenetic signal in the life span trait. On the left the histogram of null number of changes and the observed number of changes (red arrow); and on the right the phylogenetic tree with each color on the tip showing different trait states.


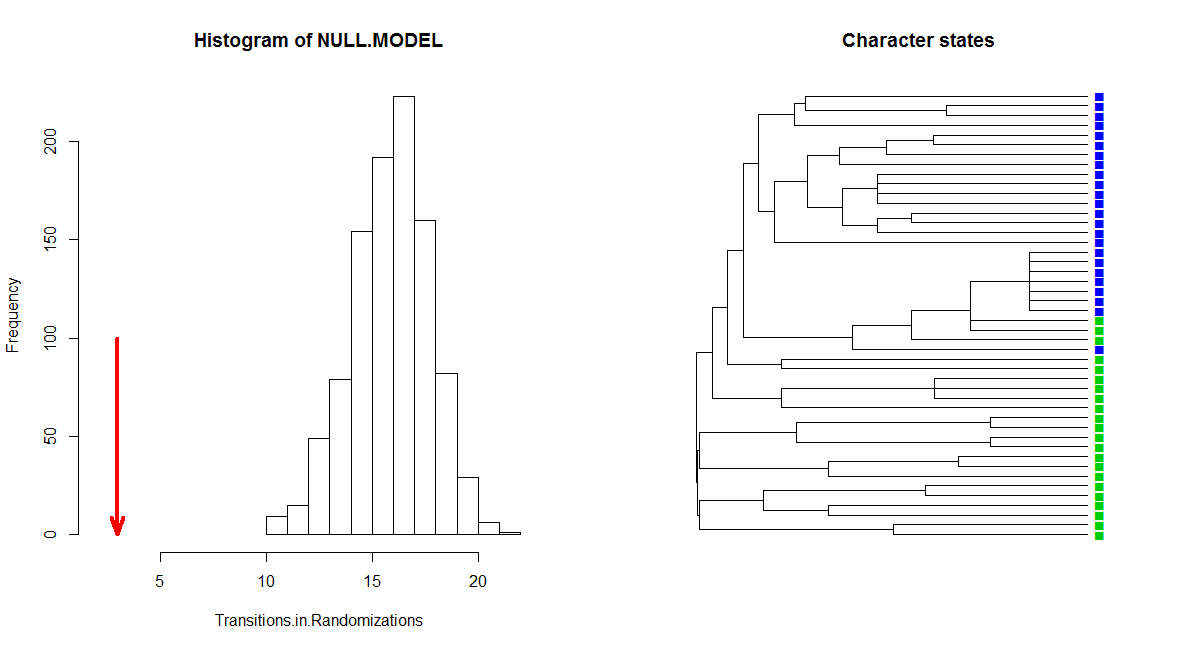


**Figure S4.** Figures showing the result of Maddison and Slatkin (1999) method for calculating phylogenetic signal in the body shape trait. On the left the histogram of null number of changes and the observed number of changes (red arrow); and on the right the phylogenetic tree with each color on the tip showing different trait states.


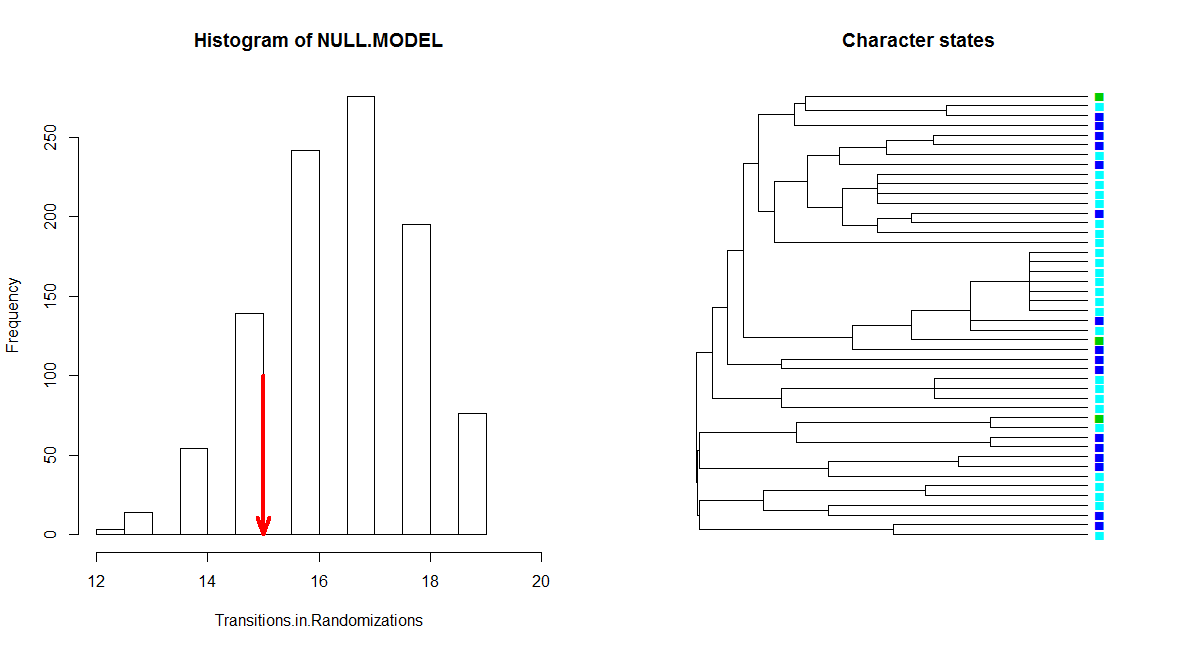


**Figure S5.** Figures showing the result of Maddison and Slatkin (1999) method for calculating phylogenetic signal in the reophily trait. On the left the histogram of null number of changes and the observed number of changes (red arrow); and on the right the phylogenetic tree with each color on the tip showing different trait states.


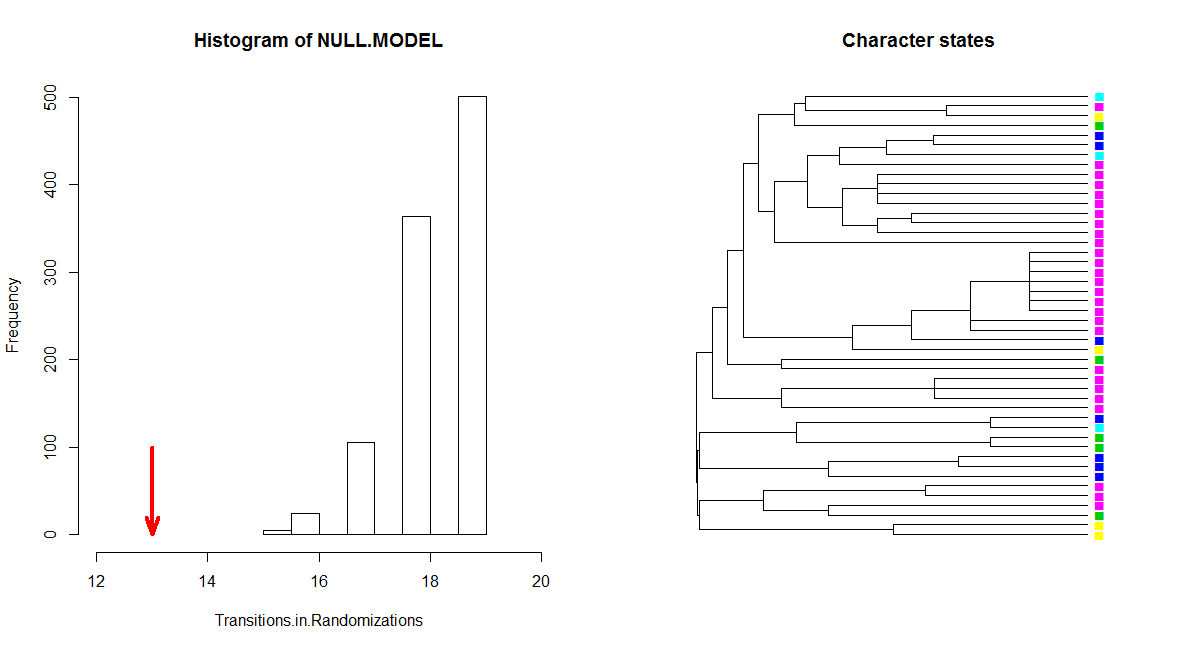


**Figure S6.** Figures showing the result of Maddison and Slatkin (1999) method for calculating phylogenetic signal in the micro-habitat preference trait. On the left the histogram of null number of changes and the observed number of changes (red arrow); and on the right the phylogenetic tree with each color on the tip showing different trait states.


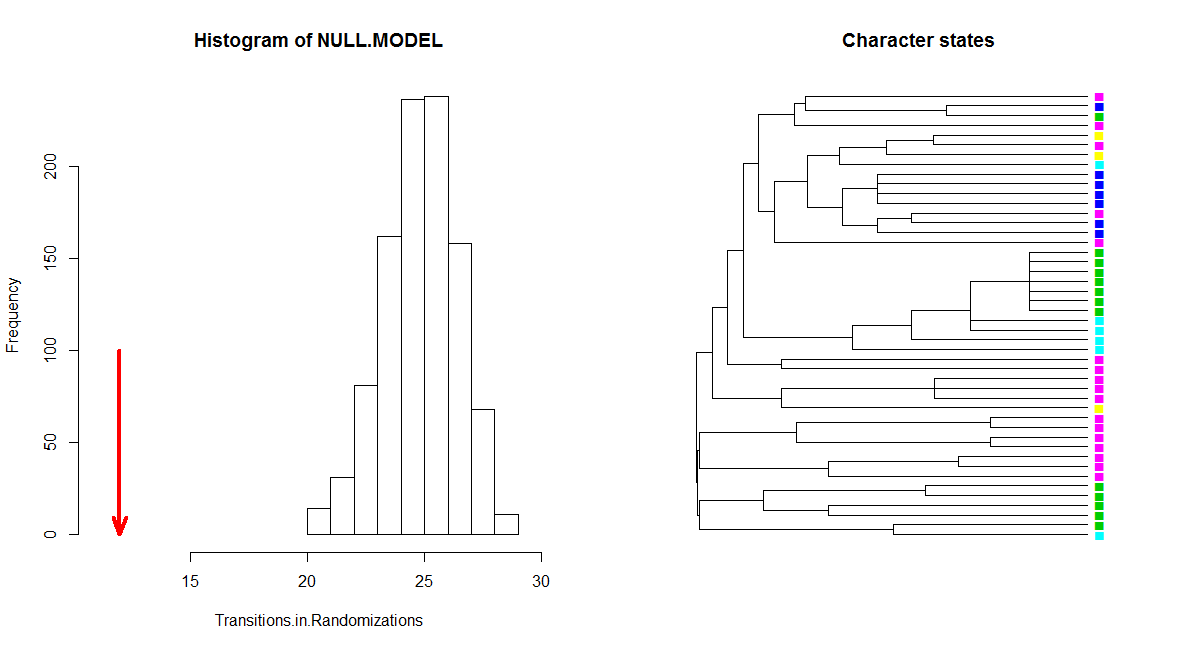


**Figure S7.** Figures showing the result of Maddison and Slatkin (1999) method for calculating phylogenetic signal in the trophic position trait. On the left the histogram of null number of changes and the observed number of changes (red arrow); and on the right the phylogenetic tree with each color on the tip showing different trait states.


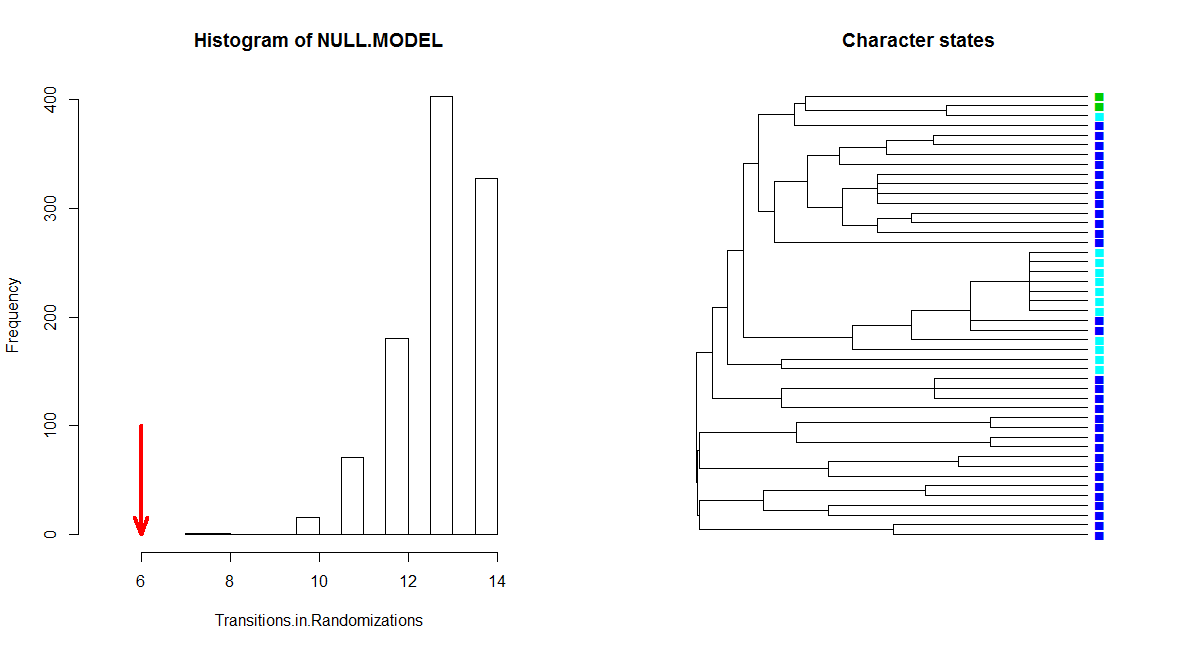


**Figure S8.** Figures showing the result of Maddison and Slatkin (1999) method for calculating phylogenetic signal in the respiration trait. On the left the histogram of null number of changes and the observed number of changes (red arrow); and on the right the phylogenetic tree with each color on the tip showing different trait states.

Among the results of phylogenetic signal the only two discordancies were for respiration and shelter. Mantel tests suggested no phylogenetic signal while Maddison and Slatkin (1991) approach resulted in significant values (*P*=0.001). Based on the high proportion of a single trait state in the species pool for these two traits (see above that 69 and 76% of species have the same trait state for respiration and shelter, respectively) we suggest that Mantel test (and other tests based on distance matrix) should have low power to detect phylogenetic signal when traits have low variability in the species pool. In these cases, tests based on evolution models, such as those based on parsimony should have more power to detect trait conservatism.

Most Mantel correlograms did not show regularly decreasing curves indicating that the evolution rates in traits was not constant throughout the phylogenetic tree. Correlations in first distance classes in several traits were similar or even lower than correlations found for species that have the common ancestor close to the root (figure S9). This means that closely related species can sometimes be less similar than more distantly related ones for several traits. Mantel correlograms thus suggests a common trait lability on the taxa under study. The distances calculated on all traits together and α niche traits showed more linear decrease than β niche traits. However, absolute correlations were low (<0.32) indicating trait lability. Correlations in first distance classes in several traits were similar or even lower than correlations found for species that have the common ancestor close to the root. This means that closely related species can sometimes be less similar than more distantly related ones for several traits. Mantel correlograms thus suggests a common trait lability on the taxa under study.


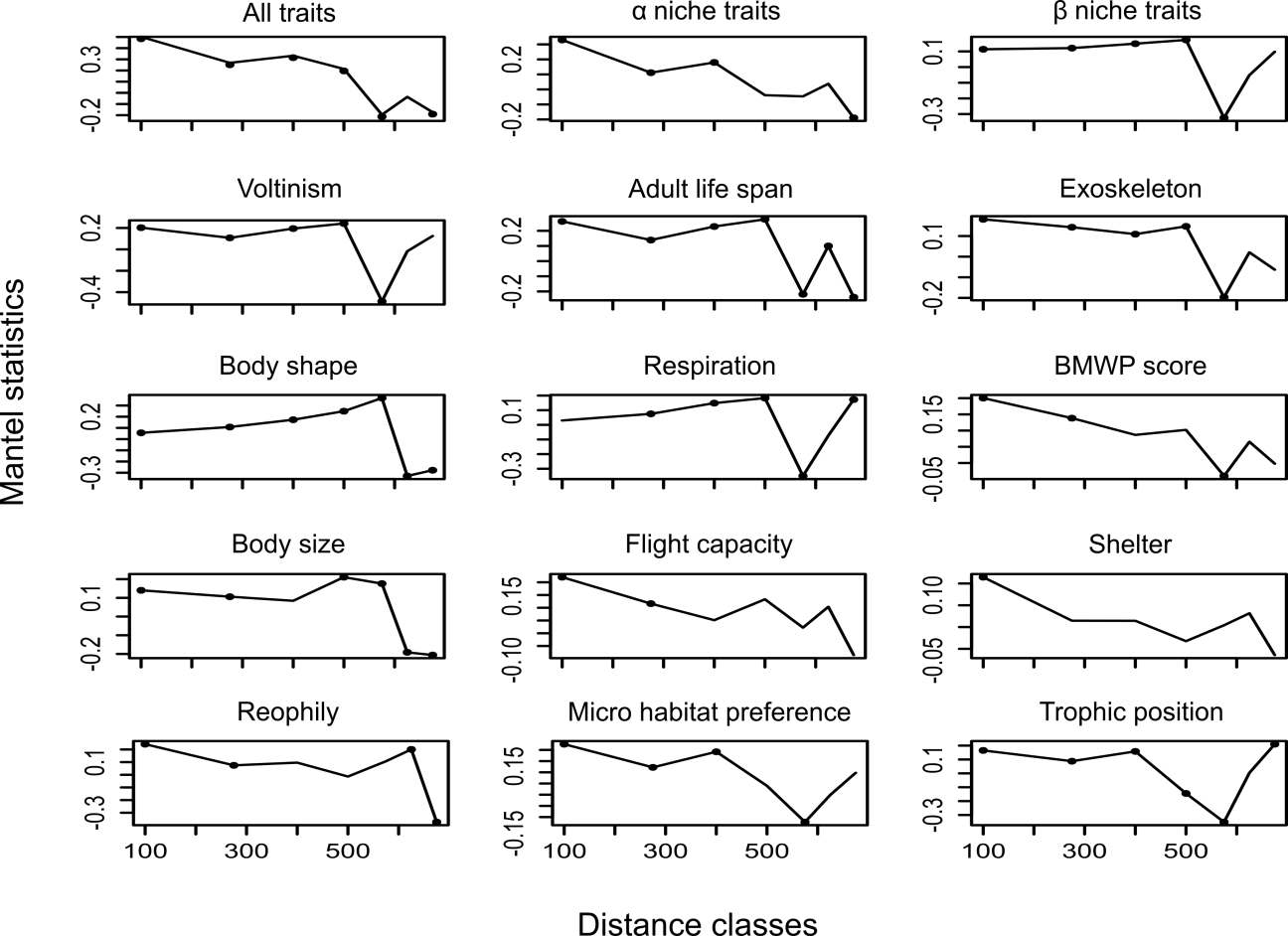


**Figure S9.** Mantel correlograms showing the correlation among trait distances and phylogenetic distances. A phylogenetic signal corresponds to a decreasing correlogram from positive correlations meaning that related genera have similar traits to negative correlations meaning that distantly related genera have distinct traits. Correlations were made for all combined traits,α and β niche traits, and for all individual traits. The phylogenetic distances among taxa were divided into classes according to the following intervals: [0,200[, [200,350[, [350,450[, [450,550[, [550,600[, [600,650[, [650,700[.Intervals relate to millions of years of divergence based on phylogenetic reconstructions (Saito et al. 2015). A distance class was then positioned on the x-axis at the center of its interval.We chose these intervals such that there were enough distances among taxa in each interval for the correlations between trait and phylogenetic distances to be estimated correctly. For a given class of phylogenetic distances, a black break point means significant (P<0.01) Mantel correlation between trait distance and a binary indicator of phylogenetic distance defined as 0 if the phylogenetic distance is within the selected distance class and 1 if it is not.

**References**

Bauer, U., Clemente, C.J., Renner, T. & Federle, W. (2012) Form follows function: morphological diversification and alternative trapping strategies in carnivorous Nepenthes pitcher plants. *Journal of Evolutionary Biology*, **25**, 90–102.

Blomberg, S.P., Garland, T. & Ives, A.R. (2003) Testing for phylogenetic signal in comparative data: behavioral traits are more labile. *Evolution*, **57**, 717–745.

Maddison, W.P. & Slatkin, M. (1991) Null models for the number of evolutionary steps in a character on a phylogenetic tree. *Evolution*, **45**, 1184–1197.

Montesinos-Navarro, A., Segarra-Moragues, J.G., Valiente-Banuet, A. & Verdú, M. (2012) Plant facilitation occurs between species differing in their associated arbuscular mycorrhizal fungi. *The New phytologist*, **196**, 835–44.

Moro, M.F., Silva, I.A., de Araújo, F.S., Nic Lughadha, E., Meagher, T.R. & Martins, F.R. (2015) The role of edaphic environment and climate in structuring phylogenetic pattern in seasonally dry tropical plant communities. *PloS one*, **10**, e0119166.

Nichols, E., Uriarte, M., Bunker, D.E., Favila, M.E., Slade, E.M., Vulinec, K., Larsen, T., Vaz-de-Mello, F.Z., Louzada, J., Naeem, S. & Spector, S.H. (2013) Trait-dependent response of dung beetle populations to tropical forest conversion at local and regional scales. *Ecology*, **94**, 180–189.

Pavoine, S. & Ricotta, C. (2012) Testing for Phylogenetic Signal in Biological Traits : the Ubiquity of Cross-Product Statistics. , 828–840.

Valiente-Banuet, A. & Verdú, M. (2007) Facilitation can increase the phylogenetic diversity of plant communities. *Ecology letters*, **10**, 1029–36.

Verdú, M. & Pausas, J.G. (2007) Fire drives phylogenetic clustering in Mediterranean Basin woody plant communities. *Journal of Ecology*, **95**, 1316–1323.

**Phylogenies and traits provide distinct insights about the historical and contemporary assembly of aquatic insect communities**

Victor S. Saito, Marcus Vinicius Cianciaruso,Tadeu Siqueira, Alaide A. Fonseca-Gessner, Sandrine Pavoine

**Supporting Information**

**Appendix S2: Supporting results from community structure analyses**

The taxa shuffle null model can be problematic when species abundance have phylogenetic signal or is strongly related to specific trait states (Hardy 2008). For that we used a test calculates a species abundance phylogenetic (or trait) clustering or overdispersion. We used APD statistic and null model 1p described by Olivier Hardy (2008). We found that only one out of 16 tests (12 traits + combined alpha traits + combined beta traits + all traits + phylogeny) was significant (6%). This is close to the nominal alpha error risk of 5%. We thus consider that the taxa shuffle null model is not biased for our analyses of community structure.

**Table S3.** Results of APD test for species abundance phylogenetic and trait clustering or overdispersion. APD index > 0 indicates ‘mean species abundance phylogenetic (or trait) clustering’, and APD index < 0 indicates ‘mean species abundance phylogenetic (or trait) overdispersion’.

| APD test applied to: | APD index | *P* value |
| --- | --- | --- |
| Phylogeny | -0.01 | 0.548 |
| All traits | 0 | 0.820 |
| α niche traits | 0.03 | 0.550 |
| β niche traits | 0.06 | 0.538 |
| Shelter | -0.32 | 0.256 |
| Exoskeleton | -0.08 | 0.248 |
| Life span | 0 | 0.978 |
| Body shape | -0.03 | 0.530 |
| Reophily | 0.16 | 0.160 |
| Microhabitat preference | 0.07 | 0.642 |
| Trophic position | -0.08 | 0.244 |
| Respiration | 0.15 | 0.506 |
| BMWP | 0 | 0.966 |
| Body size | 0.20 | 0.222 |
| Flight capacity | 0.43 | 0.006 |
| Voltinism | 0.05 | 0.816 |

Results of NRI and NTI using different species pools.

**
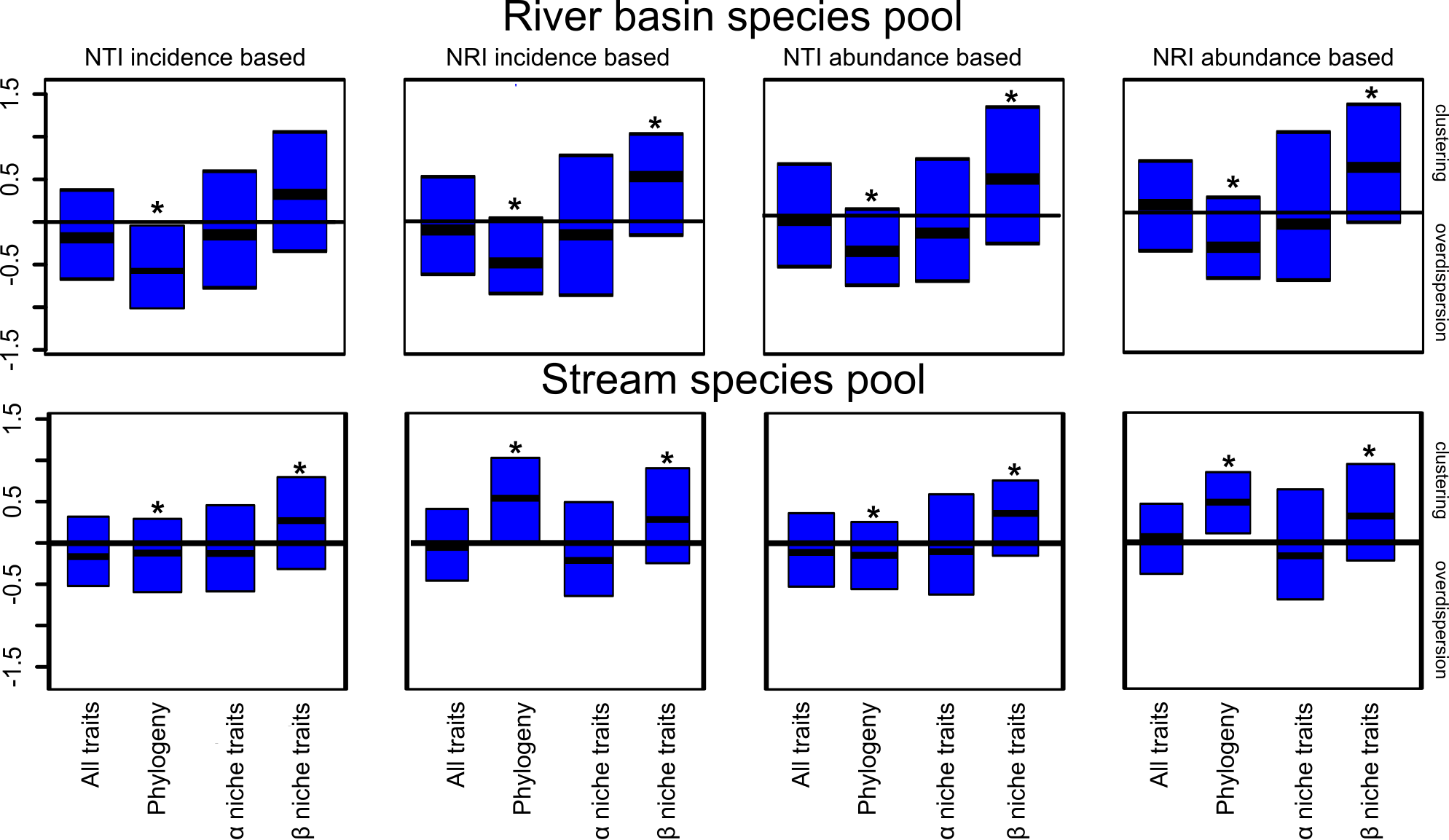
**

**Figure S10. NTI and NRI results on rifle micro-scale using the two distinct null models.** Box plots of values of Nearest taxon index (NTI) and Net relatedness index (NRI) on riffle micro-scale (n=130) calculated with trait and phylogenetic distances using two different pools of genera. The river basin pool considers all genera present in the Itanhaém river basin. The stream pool instead considers only the genera of each stream. Trait distances were calculated in three different ways: using all traits, using α niche traits and using β niche traits. Median values significantly different from zero according to two-tailed Wilcoxon test have “*” for P<0.01.

**
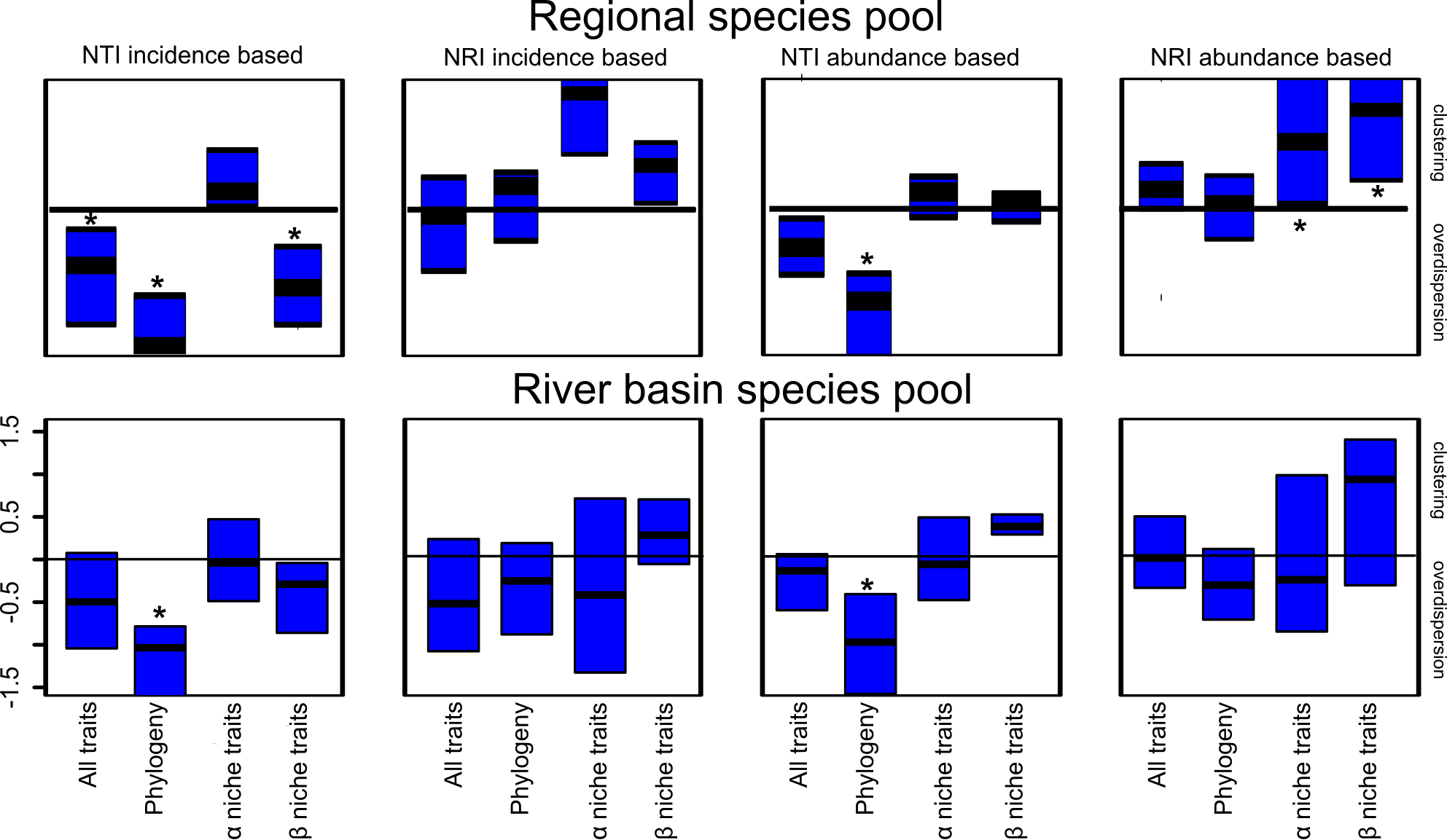
**

**Figure S11. NTI and NRI results on stream scale using the two distinct null models.** Box plots of values of Nearest taxon index (NTI) and Net relatedness index (NRI) on stream scale (n=13) calculated with trait and phylogenetic distances using two different pools of genera. The regional pool considers all genera sampled in the State of São Paulo (Suriano *et al.* 2011). The river basin pool instead considers only the genera of the Itanhaém river basin. Trait distances were calculated in three different ways: using all traits, using α niche traits and using β niche traits. Median values significantly different from zero according to two-tailed Wilcoxon test have “*” for P<0.01.

Correlation between results of NRI and NTI and SES.PD.


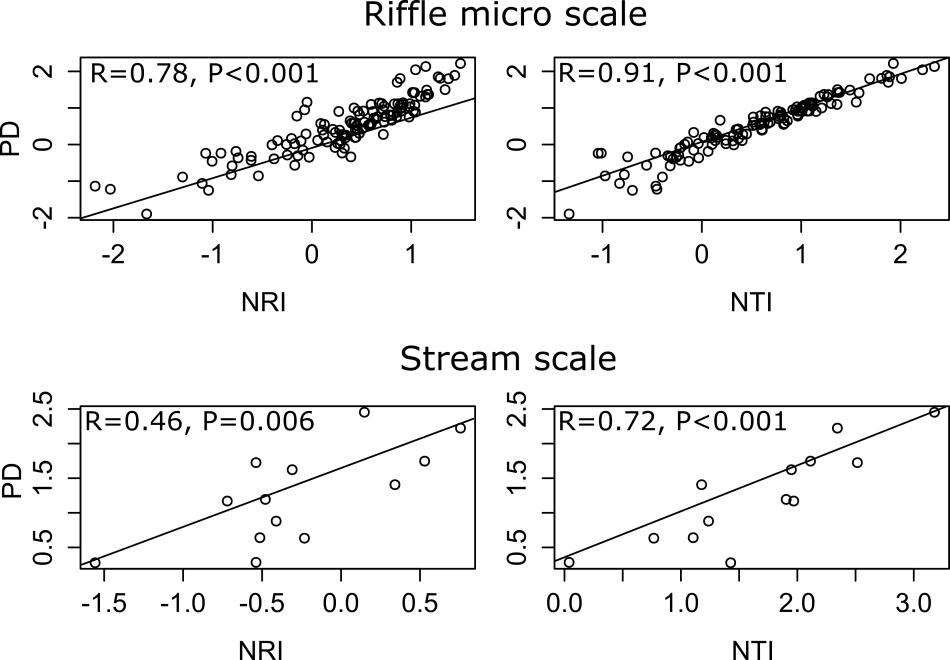


**Figure S12. Relationship between phylogenetic diversity and phylogenetic structure metrics.** Dot plot of correlation between the standardized effect size of phylogenetic diversity (PD) and the net relatedness index (NRI), and between PD and the nearest taxon index (NTI). R and P values are from a linear model.

**References**

Suriano, M.T., Fonseca-Gessner, A.A., Roque, F.O. & Froehlich, C.G. (2011) Choice of macroinvertebrate metrics to evaluate stream conditions in Atlantic Forest, Brazil. *Environmental Monitoring and Assessment*, **175**, 87–101.

Hardy, O.J. (2008) Testing the spatial phylogenetic structure of local communities: statistical performances of different null models and test statistics on a locally neutral community. *Journal of Ecology*, **96**, 914–926.

**Phylogenies and traits provide distinct insights about the historical and contemporary assembly of aquatic insect communities**

Victor S. Saito, Marcus Vinicius Cianciaruso, Tadeu Siqueira, Alaide A. Fonseca-Gessner, Sandrine Pavoine

**Supporting Information**

**Appendix S3: Additional analyses exploring the combination of traits**

*First analysis*: We ran NRI and NTI for each individual trait using incidence and abundance data in the riffle micro-scale and in the stream scale.

*
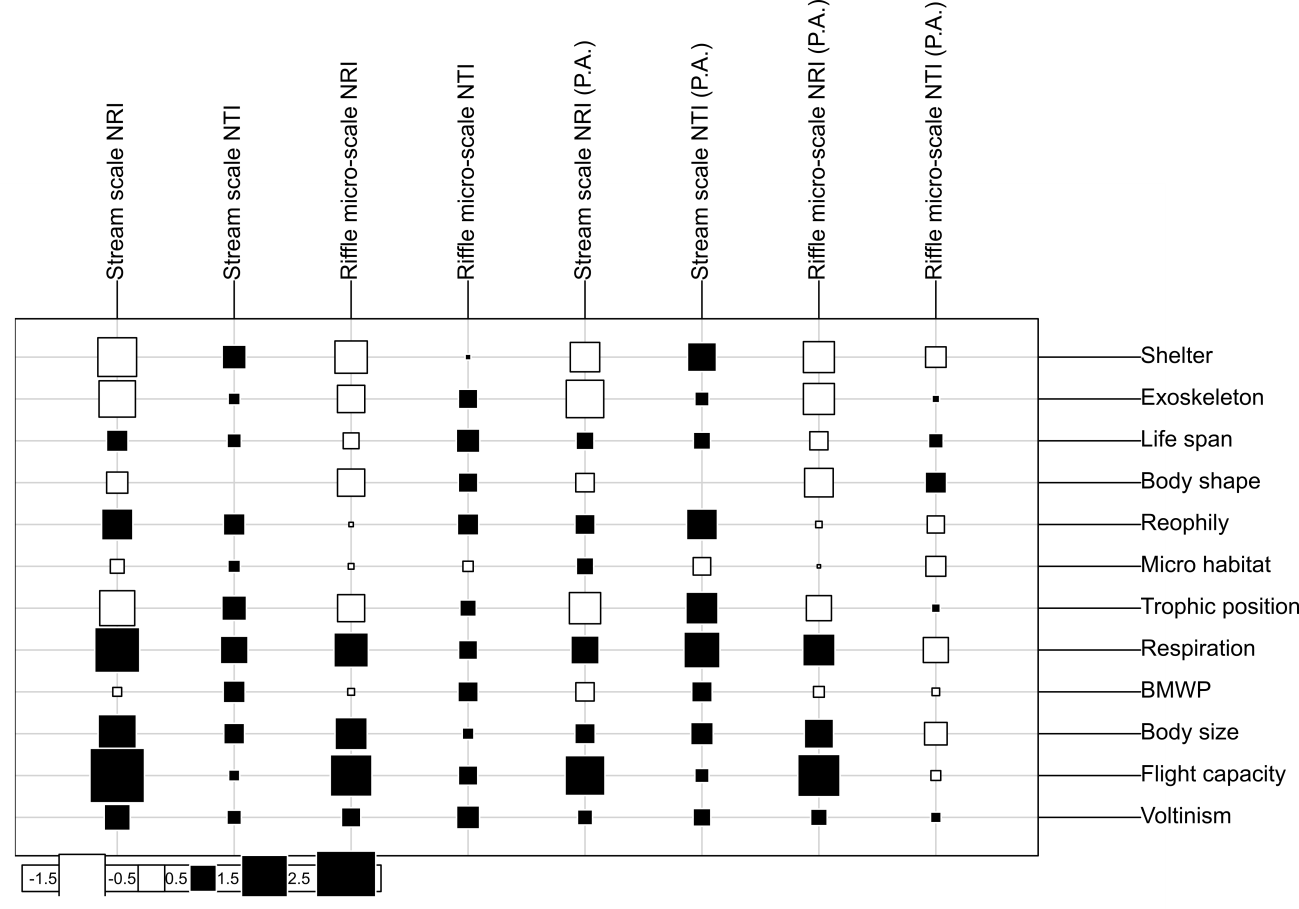
*

**Figure S13. Results of NRI and NTI for each individual trait.** Size and color of squares are related to the mean value of the indices. White squares are interpreted as mean tendency to overdispersion and black squares as mean tendency to clustering.

Similar to what we found using the redundancy analysis, we also found that the traits that resulted in strongest clustering (flight capacity) and overdispersion (exoskeleton) were not those expected to be related to the alpha and beta niche of aquatic insects. This could be explained if these traits were actually reflecting response of other linked traits (e.g. physiological or phylogenetic constraints).

*Second analysis:* We tested the correlation among traits using pairwise Mantel correlations.


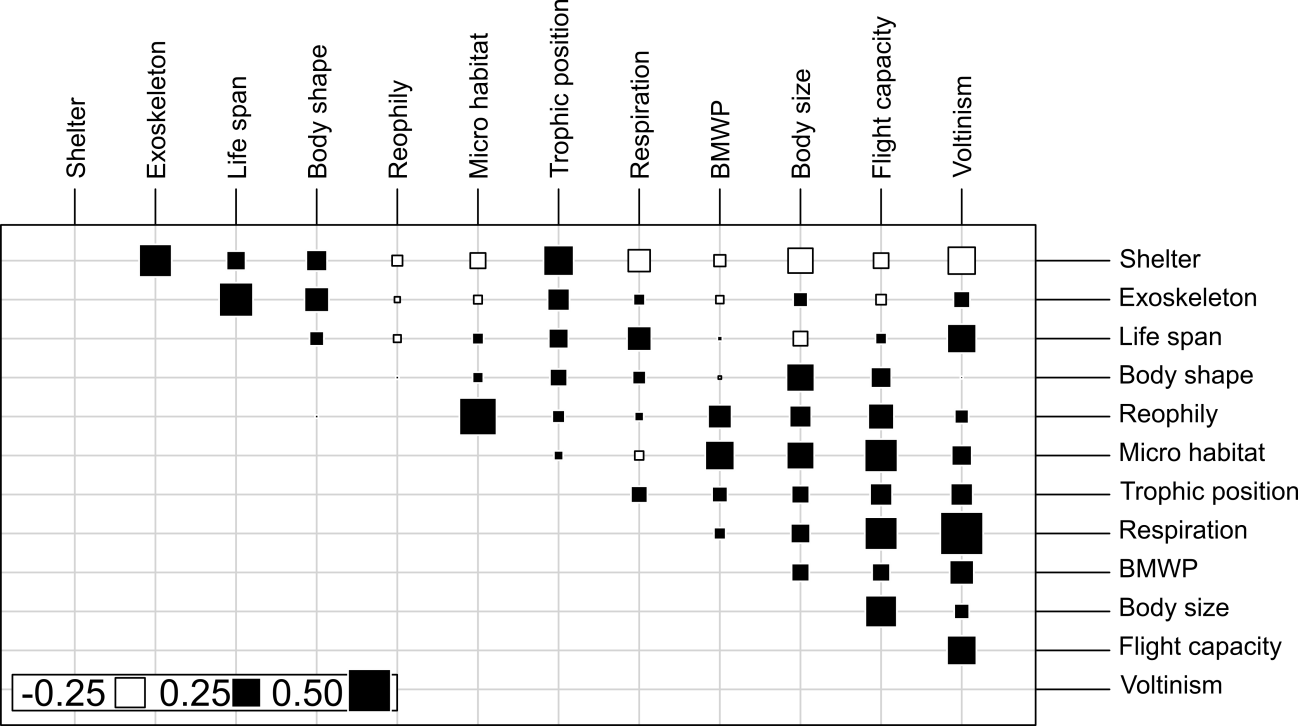


**Figure S14. The correlations among traits were tested using Mantel correlations.** Square size and color indicates the value and direction of correlation. Voltinism and respiration presented moderated correlation (r=0.52), but other traits were independent of each other (r<0.32).

These results suggest that strongest clustering and overdispersion in traits not expected to be related to the alpha and beta niche of aquatic insects were not due to linkage with other traits.

*Third analysis:* We explored the relationship between the number of traits used and the results of dispersion analysis.


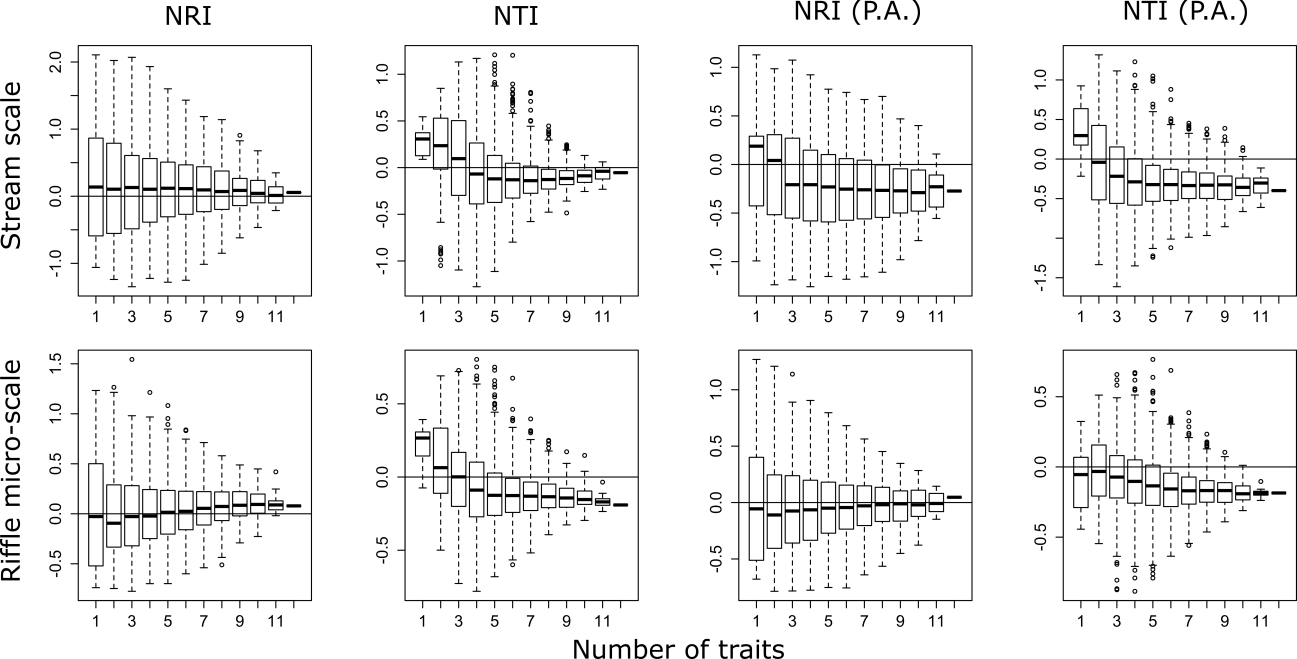


**Figure S15. Results of NRI and NTI with a varying number of traits used in distance calculations.** NRI have a decreasing variation with increasing number of traits. NTI have an increased variation with increasing number of traits but it decreased after 4-5 traits. P.A. are analysis using presence-absence data.
